# Feature Selection Using Approximate Conditional Entropy Based on Fuzzy Information Granule for Gene Expression Data Classification

**DOI:** 10.3389/fgene.2021.631505

**Published:** 2021-03-30

**Authors:** Hengyi Zhang

**Affiliations:** College of Animal Science and Technology, Northwest A&F University, Yangling, China

**Keywords:** feature selection, Laplacian kernel, fuzzy information granule, fuzzy relation matrix, approximate conditional entropy

## Abstract

Classification is widely used in gene expression data analysis. Feature selection is usually performed before classification because of the large number of genes and the small sample size in gene expression data. In this article, a novel feature selection algorithm using approximate conditional entropy based on fuzzy information granule is proposed, and the correctness of the method is proved by the monotonicity of entropy. Firstly, the fuzzy relation matrix is established by Laplacian kernel. Secondly, the approximately equal relation on fuzzy sets is defined. And then, the approximate conditional entropy based on fuzzy information granule and the importance of internal attributes are defined. Approximate conditional entropy can measure the uncertainty of knowledge from two different perspectives of information and algebra theory. Finally, the greedy algorithm based on the approximate conditional entropy is designed for feature selection. Experimental results for six large-scale gene datasets show that our algorithm not only greatly reduces the dimension of the gene datasets, but also is superior to five state-of-the-art algorithms in terms of classification accuracy.

## Introduction

The development of DNA microarray technology has brought about a large number of gene expression data. It is a hot topic in bioinformatics to analyze and mine the knowledge behind these data (Sun et al., 2019b). As the most basic data mining method, classification is widely used in the analysis of gene expression data. Due to the small sample size and high dimensionality of gene expression data, the traditional classification methods are often ineffective when applied to gene expression data directly (Fu and Wang, 2003; Mitra et al., 2011; Phan et al., 2012; Konstantina et al., 2015). It has become a consensus in the academic community to reduce the dimensionality before classification. Feature selection is the most widely used dimensionality reduction method in gene expression data because it can maintain the biological significance of each feature. Feature selection can not only reduce the time and space complexity of classification learning algorithm, avoid dimensionality disaster, and improve the prediction accuracy of classification, but also help to explain biological phenomena.

Feature selection methods are generally divided into three categories: filter, wrapper, and embedded method (Hu et al., 2018). The filter method obtains the optimal subset of features by judging the similarity between the features and the objective function based on the statistical characteristics of data. The wrapper method uses a specific model to carry out multiple rounds of training. After each round of training, several features are removed according to the score of the objective function, and then the next round of training is carried out based on the new feature set. In this way, recursion is repeated until the number of remaining features reaches the required number. The embedded method uses machine learning algorithm to get the weight coefficient of each feature in the first place, and then selects the feature according to the weight coefficient from large to small. Wrapper and embedded methods have heavy computational burden and are not suitable for large-scale gene data sets. Our feature selection method belongs to the filter method, in which a heuristic search algorithm is used to find an optimal subset of features using approximate conditional entropy based on fuzzy information granule for gene expression data classification.

Attribute reduction is a fundamental research topic and an important application of granular computing (Dong et al., 2018; Wang et al., 2019). Attribute reduction can be used for feature selection. Granular computing is a new concept and new computing paradigm of information processing, which is mainly used to deal with fuzzy and uncertain information (Qian et al., 2011).

Pawlak (1982) proposed the rough set theory. Rough set theory is a new mathematical tool to deal with fuzziness and uncertainty. Granular computing is one of the important research contents of rough set theory. On the basis of equivalence relation, rough set theory is only suitable for dealing with discrete data widely existing in real life. When dealing with attribute reduction problem of continuous data in classical rough set theory, discretization method is often used to convert continuous data into discrete data, but the discretization will inevitably lead to information loss (Dai and Xu, 2012). To overcome this drawback, Hu et al. proposed a neighborhood rough set model (Hu et al., 2008, 2011). Using neighborhood rough set model to select attribute of decision table containing continuous data can keep classification ability well and need not discretize it. The existing neighborhood rough set attribute reduction methods are based on the perspective of algebra or information theory. The definition of attribute significance based on algebra theory only describes the influence of attributes on the definite classification subset contained in the universe. The definition of attribute significance based on information theory only describes the influence of attributes on uncertain classification subsets contained in the universe. A single perspective is not comprehensive (Jiang et al., 2015).

Zadeh (1979) proposed the concept of information granulation based on fuzzy sets theory. Objects in the universe are granulated into a set of fuzzy information granules by a fuzzy-binary relation (Tsang et al., 2008; Jensen and Shen, 2009).

In this article, a heuristic feature selection algorithm based on fuzzy information granules and approximate conditional entropy is designed to improve the classification performance of gene expression data sets. The experimental results for several gene expression data sets show that the proposed algorithm can find optimal reduction sets with few genes and high classification accuracy.

The remainder of this article is organized as follows. Section “Materials and Methods” gives the gene expression datasets for the experiment and our feature selection algorithm. Section “Experimental Results and Analysis” shows and analyzes the experimental results. Section “Conclusion and Discussion” summarizes this study and discusses future research focus.

## Materials and Methods

### Gene Expression Data Sets

The following six gene expression datasets are used in this article.

(1) Leukemia1 dataset consists of 7129 genes and 72 samples with two subtypes: patients and healthy people (Sun et al., 2019a).
(2) Leukemia2 dataset consists of 5327 genes and 72 samples with three subtypes: ALL-T (acute lymphoblastic leukemia, T-cell), ALL-B (acute lymphoblastic leukemia, B-cell), and AML (acute myeloid leukemia) (Dong et al., 2018).
(3) Brain Tumor dataset consists of 10,367 genes and 50 samples with four subtypes (Huang et al., 2017).
(4) 9_Tumors dataset consists of 5726 genes and 60 samples with nine subtypes: non-small cell lung cancer, colon cancer, breast cancer, ovarian cancer, leukemia, kidney cancer, melanoma, prostate cancer, and central nervous system cancer (Ye et al., 2019).
(5) Robert dataset consists of 23,416 genes and 194 samples with two subtypes: Musculus CD8+T-cells and L1210 cells (Kimmerling et al., 2016).
(6) Ting dataset consists of 21,583 genes and 187 samples with seven subtypes: GMP cells, MEF cells, MP cells, nb508 cells, TuGMP cells, TuMP cells, and WBC cells (Ting et al., 2014).

The six gene expression datasets are summarized in Table 1.

**TABLE 1:** Description of six experimental datasets.

No.	Datasets	Genes	Samples	Classes
1	Leukemia1	7129	72	2 (47/25)
2	Leukemia2	5327	72	3 (9/38/25)
3	Brain_Tumor	10,367	50	4 (14/7/14/15)
4	9_Tumors	5726	60	9 (9/7/8/6/6/8/8/2/6)
5	Robert	23,416	194	2 (88/106)
6	Ting	21,583	187	7 (18/12/75/16/20/34/12)

### Fuzzy Sets and Fuzzy-Binary Relation

Let *U* = {*x*_1_, *x*_2_, …, *x*_*n*_} be a nonempty finite set and denote a universe, *I* = [0, 1], *I^U^* denotes all fuzzy sets on *U*.

Fuzzy sets are regarded as the extensions of classical sets (Zadeh, 1965).

*F* is a fuzzy set on *U*, i.e., *F*: *U* → *I*, then *F*(*x*_*i*_) is the membership degree of *x*_*i*_ to *F*.

The cardinality of *F* ∈ *I^U^* is $\left|F\right|=\sum_{i=1}^{n}F\left(x_{i}\right)$.

Fuzzy-binary relation are fuzzy sets on two universes. *I*^*U*×*U*^ denotes all fuzzy-binary relations on *U* × *U*.

Fuzzy-binary relation *R* can be represented by

(1) $$M_{R}=\left(\begin{array}{cccc} r_{11} & r_{12} & \cdots & r_{1n}\\ r_{21} & r_{22} & \cdots & r_{2n}\\ \cdots & \cdots & \cdots & \cdots \\ r_{n1} & r_{n2} & \cdots & r_{nn} \end{array}\right)$$

where *r*_*ij*_ = *R*(*x*_*i*_, *x*_*j*_) ∈ *I* is the similarity of *x*_*i*_ and *x*_*j*_.

### Information Systems and Rough Sets

**Definition 2.1** (Li et al., 2017). Let *U*be a set of objects and *A* a set of attributes. Suppose that *U* and *A* are finite sets. If each attribute *a* ∈ *A* determines an information function *a*:*U*→*V*_*a*_, where *V*_*a*_ is the set of function values of attribute *a*, then the pair (*U*, *A*) is called an information system.

Moreover, if *A* = *C*⋃*D*, *C* is a condition attribute set and *D* is a decision attribute set, then the pair (*U*, *A*) is called a decision information system.

If (*U*, *A*) is an information system and *P* ⊆ *A*, then an equivalence relation (or indiscernibility relation) *ind*(*P*) can be defined by (*x*, *y*) ∈ *ind*(*P*)⇔∀*a* ∈ *P*, *a*(*x*) = *a*(*y*).

Obviously, $ind\left(P\right)=\underset{a\in P}{⋂}ind\left(\left\{a\right\}\right)$.

For *P* ⊆ *A* and *x* ∈ *U*, denote [*x*]_*ind*(*P*)_ = {*y*|(*x*, *y*) ∈ *ind*(*P*)} and *U*/*ind*(*P*) = {[*x*]_*ind*(*P*)_|*x*∈*U*}.

Usually, *[x]*_*ind(P)*_ and *U*/*ind*(*P*) are briefly denoted by *[x]*_*P*_ and *U*/*P*, respectively.

According to the rough set theory, for *P* ⊆ *A*, *X* ⊆ *U* is characterized by $\bar{P}\left(X\right)$ and $\underline{P}\left(X\right)$, where $\underline{P}\left(X\right)=⋃\left\{Y|Y\in U/P,Y\subseteq X\right\}$ and $\bar{P}\left(X\right)=⋃\left\{Y|Y\in U/P,Y⋂X\neq \phi \right\}$.

$\underline{P}\left(X\right)$ and $\bar{P}\left(X\right)$ are referred to as the lower and upper approximations of *X*, respectively.

*X* is crisp if $\bar{P}\left(X\right)=\underline{P}\left(X\right)$ and *X* is rough if $\bar{P}\left(X\right)\neq \underline{P}\left(X\right)$.

### The Approximately Equal Relation on Fuzzy Sets

Given *F*,*G* ∈ *I^U^*. For *x* ∈ *U*, *F*(*x*) and *G*(*x*) are the membership degrees of *x* belonging to fuzzy sets *F* and *G*, respectively. *F*(*x*) and *G*(*x*) ∈ [0,1]. Actually, it is very difficult to ensure that the equation *F*(*x*) = *G*(*x*) holds. For this reason, we propose the following approximately equal relation of fuzzy sets.

**Definition 2.2** Given *A*,*B* ∈ *I^U^*. If there exists *k* ∈ *N*(*k*≥2) such that for any *x* ∈ *U*, *A*(*x*),*B*(*x*) ∈ [0,1/*k*) or *A*(*x*),*B*(*x*) ∈ [1/*k*,2/*k*)…or *A*(*x*),*B*(*x*) ∈ [(*k*−1)/*k*,1], then we say that *A* is approximately equal to *B*, and denote it by $A\overset{k}{\approx }B$, where *k* is regarded as a threshold value.

**Definition 2.3** For each *a* ∈ *U*, define *x^R^*:*U*→[0,1],*x^R^*(*a*) = *R*(*x*,*a*)(*x* ∈ *U*), *x^R^* is referred to as a fuzzy set that means the membership degree of *a* to *x*.

**Definition 2.4** ${\left[x\right]}_{R}=\left\{y|x^{R}\left(a\right)\overset{k}{\approx }y^{R}\left(a\right),y\in U\right\}$, *[x]*_*R*_is referred to as the fuzzy equal class of *x* induced by the fuzzy relation *R* on *U*.

**Definition 2.5 [*x*_*i*_]_*R*_(*i* = 1,2,…,|*U*|)** is named as the fuzzy information granule induced by the fuzzy relation *R* on *U*.

**Definition 2.6***G*(*R*) = {[*x*_1_]_*R*_,[*x*_2_]_*R*_,…,[*x*_*n*_]_*R*_} is referred to as the fuzzy-binary granular structure of the universe *U* induced by *R*.

It is easy to prove: $\underline{P}\left(X\right)=\left\{x|{\left[x\right]}_{R}\subseteq X,{\left[x\right]}_{R}\in G\left(R\right)\right\}$, $\bar{P}\left(X\right)=\left\{x|{\left[x\right]}_{R}⋂X\neq \phi ,{\left[x\right]}_{R}\in G\left(R\right)\right\}$.

### Fuzzy-Binary Relation Based on Laplacian Kernel

Hu et al. (2010) found that there are some relationships between rough sets and Gaussian kernel method, so Gaussian kernel is used to obtain fuzzy relations. Compared with Gaussian kernel, Laplacian kernel has higher peak, faster reduction and smoother tail. Therefore, Laplacian kernel is better than Gaussian kernel in describing the similarity between objects. In this article, we use Laplacian kernel $k\left(x_{i},x_{j}\right)=\exp\left(-\frac{||x_{i}-x_{j}||}{\sigma }\right)$ to extract the similarity between two objects from decision information system, where ||*x*_*i*_−*x*_*j*_|| is the Euclidean distance between two objects *x*_*i*_ and *x*_*j*_. In general, σ is a given positive value.

Obviously, *k*(*x*_*i*_, *x*_*j*_) satisfies:

(1) *k*(*x*_*i*_, *x*_*j*_)∈(0,1].
(2) *k*(*x*_*i*_, *x*_*j*_) = *k*(*x*_*j*_,*x*_*i*_).
(3) *k*(*x*_*i*_, *x*_*i*_) = 1.

Let *R* = (*k*(*x*_*i*_, *x*_*j*_))_*n*×*n*_, then *R* is called the fuzzy relation matrix induced by Laplacian kernel.

### Feature Selection Using Approximate Conditional Entropy Based on Fuzzy Information Granule

#### Approximate Accuracy and Approximate Conditional Entropy

**Definition 2.7** Given a decision information system (*U*, *C*⋃*D*), ∀*X* ⊆ *U*, *X* ≠ ϕ (ϕ is an empty set), then the approximate accuracy of *X* is defined as

(2) $$a\left(X\right)=\frac{\left|\underline{P}\left(X\right)\right|}{\left|\bar{P}\left(X\right)\right|}$$

where |.| denotes the cardinality of set. Obviously, 0≤*a*(*X*)≤1.

**Definition 2.8** Given a decision information system (*U*,*C*⋃*D*), ∀*B* ⊆ *C*, the fuzzy information granule of object *x* under *B* is *[x]*_*R_B*_, the partition of *U* derived from *D* is {*X*_1_,*X*_2_,…,*X*_*k*_}, then the conditional entropy of *D* relative to *B* is defined as

(3) $$H\left(D/B\right)=-\sum_{j=1}^{k}\sum_{i=1}^{\left|U\right|}\frac{\left|{\left[x_{i}\right]}_{R_{B}}⋂X_{j}\right|}{\left|U\right|}\log\frac{\left|{\left[x_{i}\right]}_{R_{B}}⋂X_{j}\right|}{\left|{\left[x_{i}\right]}_{R_{B}}\right|}$$

where *R*_*B*_ denotes the fuzzy relation based on attribute set *B* and *log* is a base-2 logarithm.

The approximate accuracy can effectively measure the imprecision of the set caused by the boundary region, while the conditional entropy can effectively measure the knowledge uncertainty caused by the information granularity. We combine the two to propose approximate conditional entropy.

**Definition 2.9** Let (*U*,*C*⋃*D*) be a decision information system, ∀*B* ⊆ *C*, the fuzzy information granule of object *x* under *B* is *[x]*_*R_B*_, the partition of *U* derived from *D* is {*X*_1_,*X*_2_,…,*X*_*k*_}, *a*_*B*_(*X*_*i*_) is the approximate accuracy of *X*_*i*_ under *R*_*B*_, then the approximate conditional entropy of *D* relative to *B* is defined as

$$H_{ace}\left(D/B\right)=-\sum_{j=1}^{k}\sum_{i=1}^{\left|U\right|}\log\left(2-a_{B}\left(X_{j}\right)\right)\frac{\left|{\left[x_{i}\right]}_{R_{B}}⋂X_{j}\right|}{\left|U\right|}$$

(4) $$\log\frac{\left|{\left[x_{i}\right]}_{R_{B}}⋂X_{j}\right|}{\left|{\left[x_{i}\right]}_{R_{B}}\right|}$$

**Theorem 2.1** Let (*U*,*C*⋃*D*) be a decision information system, ∀*B* ⊆ *C*, the fuzzy information granule of object *x* under *B* is *[x]*_*R_B*_, the partition of *U* derived from *D* is {*X*_1_,*X*_2_,…,*X*_*k*_}.

(1) *H*_*ace*_(*D*/*B*) gets the maximum value |*U*|*log*⁡|*U*| if and only if [*x*_*i*_]_*R*_*B*__ = *U*(*i* = 1,2,…,*n*) and |*X*_*j*_| = 1(*j* = 1,2,…,*k* = *n*).
(2) *H*_*ace*_(*D*/*B*)gets the minimum value *0* if and only if [*x*_*i*_]_*R*_*B*__ ⊆ [*x*_*i*_]_*R*_*D*__(*i* = 1,2,…,*n*).

**Proof.** (1) Due to [*x*_*i*_]_*R*_*B*__ = *U*(*i* = 1,2,…,*n*) and |*X*_*j*_| = 1(*j* = 1,2,…,*k*), we have *a*_*B*_(*X*_*j*_) = 0(j = 1,2,…,k) according to Definition 2.7.

Thus, *log*(2−*a*_*B*_(*X*_*j*_)) = 1(*j* = 1,2,…,*k*).

Clearly, $\frac{\left|{\left[x_{i}\right]}_{R_{B}}⋂X_{j}\right|}{\left|U\right|}\log\frac{\left|{\left[x_{i}\right]}_{R_{B}}⋂X_{j}\right|}{\left|{\left[x_{i}\right]}_{R_{B}}\right|}=\frac{1}{\left|U\right|}\log\frac{1}{\left|U\right|}$.

By Definition 2.9, we have *H*_*ace*_(*D*/*B*) = |*U*|*log*⁡|*U*|.

The converse is also true.

(2) Due to [*x*_*i*_]_*R*_*B*__ ⊆ [*x*_*i*_]_*R*_*D*__(*i* = 1,2,…,*n*), we have *a*_*B*_(*X*_*j*_) = 1(j = 1,2,…,k) according to Definition 2.7. Thus *log*(2−*a*_*B*_(*X*_*j*_)) = 0(j = 1,2,..,k). Obviously, *H*_*ace*_(*D*/*B*) = 0 according to Definition 2.9.

The converse is also true.

**Theorem 2.2** Let (*U*,*C*⋃*D*) be a decision information system, ∀*L*,*M* ⊆ *C*, if *M* ⊆ *L*, then *H*_*ace*_(*D*/*M*)≥*H*_*ace*_(*D*/*L*).

**Proof.** Due to *M* ⊆ *L* ⊆ *C*, we have $\underline{P_{M}}\left(X\right)\subseteq \underline{P_{L}}\left(X\right)$ and $\bar{P_{M}}\left(X\right)⊇\bar{P_{L}}\left(X\right)$.

Then *a*_*M*_(*X*)≤*a*_*L*_(*X*) according to Definition 2.7.

By *M* ⊆ *L* and *U*/*D* = {*X*_1_,*X*_2_,…,*X*_*k*_}, we have

$$-\frac{\left|{\left[x_{i}\right]}_{R_{M}}⋂X_{j}\right|}{\left|U\right|}\log\frac{\left|{\left[x_{i}\right]}_{R_{M}}⋂X_{j}\right|}{\left|{\left[x_{i}\right]}_{R_{M}}\right|}$$

(5) $$\geq -\frac{\left|{\left[x_{i}\right]}_{R_{L}}⋂X_{j}\right|}{\left|U\right|}\log\frac{\left|{\left[x_{i}\right]}_{R_{L}}⋂X_{j}\right|}{\left|{\left[x_{i}\right]}_{R_{L}}\right|}\geq 0$$

Consequently, *H*_*ace*_(*D*/*M*)≥*H*_*ace*_(*D*/*L*) according to Definition 2.9.

Theorem 2.2 shows that *H*_*ace*_(*D*/*B*) decreases monotonically with the increase of the number of attributes in *B*, which is very important for constructing forward greedy algorithm of attributes reduction.

**Definition 2.10** Let (*U*,*C*⋃*D*) be a decision information system and *B* ⊆ *C*, if *H*_*ace*_(*D*/*B*) = *H*_*ace*_(*D*/*C*) and *H*_*ace*_(*D*/(*B*−{*b*})) > *H*_*ace*_(*D*/*C*)(∀*b* ∈ *B*), then *B* is called a reduction of *C* relative to *D*.

The first condition guarantees that the selected attribute subset has the same amount of information as the whole attribute set. The second condition guarantees that there is no redundancy in the attribute reduction set.

**Definition 2.11** Assume that (*U*,*C*⋃*D*) be a decision information system, ∀*c* ∈ *C*, define the following indicator,

(6) $$IIA\left(c,C,D\right)=H_{ace}\left(D/\left(C-\left\{c\right\}\right)\right)-H_{ace}\left(D/C\right)$$

then *IIA*(*c*,*C*,*D*) is called the importance of internal attribute of *c* in *C* relative to *D*.

**Definition 2.12** Assume that (*U*,*C*⋃*D*) be a decision information system, ∀*c* ∈ *C*, if *IIA*(*c*,*C*,*D*) > 0, then attribute *c* is called a core attribute of *C* relative to *D*.

**Definition 2.13** Assume that (*U*,*C*⋃*D*) be a decision information system, *B* ⊆ *C*, ∀*d* ∈ *C*−*B*, define the following indicator,

(7) $$IEA\left(d,B,C,D\right)=H_{ace}\left(D/B\right)-H_{ace}\left(D/\left(B⋃\left\{d\right\}\right)\right)$$

then *IEA*(*d*,*B*,*C*,*D*) is called the importance of external attribute of *d* to *B* relative to *D*.

*IEA*(*d*,*B*,*C*,*D*) shows the change of approximate conditional entropy after adding attribute *d*. The larger *IEA*(*d*,*B*,*C*,*D*) is, the more important *d* is to *B* relative to *D*.

#### Feature Selection Algorithm Using Approximate Conditional Entropy

In this article, a novel feature selection algorithm using approximate conditional entropy (FSACE) is proposed and described as follows.

**Table:** 

|
Input: A decision information system (*U*,*C*⋃*D*) and σ.
Output:A selected gene subset *B*.
Step 1. Initialize *B* = ϕ.
Step 2. Compute *H*_*ace*_(*D*/*C*).
Step 3.∀*c* ∈ *C*, compute *IIA*(*c*,*C*,*D*), if *IIA*(*c*,*C*,*D*) > 0, then *B* = *B*⋃{*c*}.
Step 4. If *B* = ϕ, then turn to step 5. If *B*≠ϕ, compute *H*_*ace*_(*D*/*B*). If *H*_*ace*_(*D*/*B*) = *H*_*ace*_(*D*/*C*), then turn to step 6; otherwise, turn to step 5.
Step 5. Let *M* = *C*−*B*, select a attribute *m* ∈ *M* so that it satisfies $IEA\left(m,B,C,D\right)=\max_{x\in M}IEA\left(x,B,C,D\right)$. Let *B* = *B*⋃{*m*}, compute *H*_*ace*_(*D*/*B*). If *H*_*ace*_(*D*/*B*) = *H*_*ace*_(*D*/*C*), then turn to step 6; otherwise, turn to step 5.
Step 6. The feature selection subset *B* is obtained, andthe algorithm ends.

## Experimental Results and Analysis

All experiments are performed on a personal computer running Windows 10 with an Intel(R) Core(TM) i7-4790 CPU operating at 3.60 GHz with 8 GB memory using MATLAB R2019a. The classifiers (KNN, CART, and SVM) are selected to verify the classification accuracy, where the parameter *k=3* in KNN and Gaussian kernel function is selected in SVM. Other parameters of the three algorithms are the default values of the software.

### Influence of Different Values of σ on Classification Performance

In this part, the classification accuracy of different Laplacian kernel parameters values of **σ** is tested. For gene expression data, feature selection aims to improve classification accuracy by eliminating redundant genes. The different values of **σ** influence the size of granulated gene data, which affects the classification accuracy of selected genes. Therefore, the different values of **σ** should be set in the process of feature selection of gene expression data sets. Moreover, the different values of **σ** also affect the composition of the selected gene subset. To obtain a suitable **σ** and a good gene subset, the classification accuracy of the selected gene subset for different values of **σ** should be discussed in detail.

**FIGURE 1:**
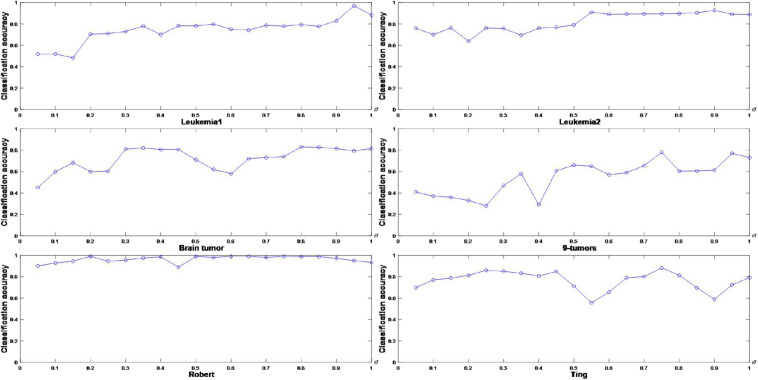
Classification accuracy for six gene expression data sets with different values of **σ**.

The corresponding experiments are performed to graphically illustrate the classification accuracy of FSACE under different values of **σ**. The results are shown in Figure 1, where the horizontal axis denotes σ ∈ [0.05,1] at intervals of 0.05, and the vertical axis represents the classification accuracy.

Figure 1 shows that **σ** greatly influences the classification performance of FSACE. **σ** is usually set to make the classification accuracy highest. Thus, the appropriate parameter values of **σ** can be obtained for each data set from Figure 1. In Figure 1A, for Leukemia1 data set, when **σ** is 0.95, the classification accuracy is the highest. In Figure 1B, for Leukemia2 data set, when **σ** is 0.55, the classification accuracy is the highest. In Figure 1C, for Brain tumor data set, when **σ** is 0.80, the classification accuracy is the highest. In Figure 1D, for 9-tumors data set, when **σ** is 0.75, the classification accuracy is the highest. In Figure 1E, for Robert data set, when **σ** is 0.60, the classification accuracy is the highest. In Figure 1F, for Ting data set, when **σ** is 0.75, the classification accuracy is the highest. Therefore, the appropriate values of **σ** for different data sets are determined.

### The Feature Selection Results and Classification Performance of FSACE

The classification results obtained from the three classifiers (KNN, CART, and SVM) with 10-fold cross-validation are shown in Table 2 on the test data by FSACE.

**TABLE 2:** Classification results of six gene expression data sets.

Data sets	Original data				Feature selection data using FSACE			
	Genes	CART	KNN	SVM	Genes	CART	KNN	SVM
Leukemia1	7129	0.822	0.839	0.917	9	0.911	0.947	0.931
Leukemia2	5327	0.849	0.820	0.834	9	0.891	0.894	0.878
Brain tumor	10,367	0.571	0.604	0.737	5	0.743	0.631	0.614
9-tumors	5726	0.273	0.349	0.334	2	0.318	0.359	0.355
Robert	23,416	0.947	0.928	0.933	14	0.985	0.974	0.990
Ting	21,583	0.864	0.826	0.841	17	0.873	0.847	0.882
Average	12,258	0.721	0.728	0.766	9.333	0.787	0.775	0.775

Table 2 shows that FSACE not only greatly reduces the dimensionality of all six gene expression data sets, but also improves the classification accuracy.

The results of feature genes selection from six gene expression data sets are shown in Table 3 using FSACE.

**TABLE 3:** The selected feature genes on six gene expression data sets using FSACE.

Data sets	The selected feature gene subsets
Leukemia1	(758,1144,1630,2659,3897,4196,5552,6471,6584)
Leukemia2	(568,848,861,1610,2197,3256,3358,4688,5032)
Brain tumor	(642,7169,7844,9413,9794)
9-tumors	(1677,2590)
Robert	(12883,1600,9892,16398,8720,4510,18137,2320,14931, 14679,10352,12481,18034,406)
Ting	(4754,5676,2503,5379,3304,4752,6015,2193,15687,641, 7938,2629,6837,4653,19016,8621,4267)

### Comparison of the Classification Performance of Several Entropy-Based Feature Selection Algorithms

To evaluate the performance of FSACE in terms of classification accuracy, FSACE algorithm is compared with several state-of-the-art feature selection algorithms, including EGGS (Chen et al., 2017), EGGS-FS (Yang et al., 2016), MEAR (Xu et al., 2009), Fisher (Saqlain et al., 2019), and Lasso (Tibshirani, 1996). According to the change trend of Fisher scores of six gene datasets, we select the top-200 genes as the reduction set for Fisher algorithm.

Tables 4–9 show the experimental results of six gene expression data sets using six different feature selection methods.

**TABLE 4:** Classification accuracy of Leukemia1 using six different feature selection algorithms.

Feature selection method	Genes	CART	KNN	SVM	Average
ECGS (Li et al., 2017)	8	0.744	0.619	0.813	0.725
EGGS-FS (Hu et al., 2010)	5	0.821	0.794	0.701	0.772
MEAR (Chen et al., 2017)	3	0.939	0.919	0.925	0.928
Fisher (Saqlain et al., 2019)	200	0.639	0.857	0.778	0.758
Lasso (Tibshirani, 1996)	52	0.857	0.960	0.972	0.929
FSACE	9	0.911	0.947	0.931	0.930

**TABLE 5:** Classification accuracy of Leukemia2 using six different feature selection algorithms.

Feature selection method	Genes	CART	KNN	SVM	Average
ECGS (Li et al., 2017)	3	0.571	0.509	0.557	0.546
EGGS-FS (Hu et al., 2010)	2	0.907	0.871	0.874	0.884
MEAR (Chen et al., 2017)	5	0.903	0.829	0.872	0.868
Fisher (Saqlain et al., 2019)	200	0.726	0.803	0.846	0.792
Lasso (Tibshirani, 1996)	37	0.817	0.914	0.909	0.880
FSACE	9	0.891	0.894	0.878	0.888

**TABLE 6:** Classification accuracy of Brain tumor using six different feature selection algorithms.

Feature selection method	Genes	CART	KNN	SVM	Average
ECGS (Li et al., 2017)	9	0.515	0.491	0.544	0.517
EGGS-FS (Hu et al., 2010)	5	0.388	0.490	0.531	0.470
MEAR (Chen et al., 2017)	–	–	–	–	–
Fisher (Saqlain et al., 2019)	200	0.630	0.704	0.617	0.650
Lasso (Tibshirani, 1996)	–	–	–	–	–
FSACE	5	0.743	0.631	0.614	0.663

**TABLE 7:** Classification accuracy of 9-tumors using six different feature selection algorithms.

Feature selection method	Genes	CART	KNN	SVM	Average
ECGS (Li et al., 2017)	1	0.177	0.102	0.672	0.317
EGGS-FS (Hu et al., 2010)	1	0.224	0.203	0.393	0.273
MEAR (Chen et al., 2017)	–	–	–	–	–
Fisher (Saqlain et al., 2019)	200	0.249	0.335	0.414	0.333
Lasso (Tibshirani, 1996)	27	0.199	0.361	0.322	0.294
FSACE	2	0.318	0.359	0.355	0.344

**TABLE 8:** Classification accuracy of Robert using six different feature selection algorithms.

Feature selection method	Genes	CART	KNN	SVM	Average
ECGS (Li et al., 2017)	11	0.948	0.937	0.964	0.950
EGGS-FS (Hu et al., 2010)	6	0.957	0.954	0.975	0.962
MEAR (Chen et al., 2017)	–	–	–	–	–
Fisher (Saqlain et al., 2019)	200	0.976	0.990	0.989	0.985
Lasso (Tibshirani, 1996)	21	0.984	0.991	0.989	0.988
FSACE	14	0.993	0.991	0.985	0.990

**TABLE 9:** Classification accuracy of Ting using six different feature selection algorithms.

Feature selection method	Genes	CART	KNN	SVM	Average
ECGS (Li et al., 2017)	12	0.793	0.781	0.651	0.742
EGGS-FS (Hu et al., 2010)	9	0.745	0.717	0.626	0.696
MEAR (Chen et al., 2017)	–	–	–	–	–
Fisher (Saqlain et al., 2019)	200	0.833	0.779	0.770	0.794
Lasso (Tibshirani, 1996)	56	0.833	0.833	0.845	0.837
FSACE	17	0.833	0.833	0.872	0.846

As shown in Tables 4, 5, FSACE has the highest average classification accuracy for Leukemia1 and Leukemia2, and exhibits better classification performance than the other five algorithms.

As shown in Tables 6, 7, MEAR cannot work on Brain Tumor data set and 9-tumors data set, its results are denoted by the sign –. FSACE obtains the highest average classification accuracy among the five feature selection algorithms for Brain Tumor data set and 9-tumors data set.

Tables 8, 9 shows that MEAR still can not work on Robert data set and Ting data set, which indicates that the algorithm is not stable. Our algorithm still has the highest classification accuracy among all the algorithms. Although the classification accuracy of our algorithm is only a little higher than lasso algorithm, the number of attributes reduced by our algorithm is much less than lasso algorithm.

Tables 4–9 show that the average number of attributes reduced by our algorithm is slightly more than that of MEAR, ECGS, and EGGS-FS, but the average classification accuracy is much higher than that of these three algorithms.

Therefore, FSACE can not only effectively remove noise and redundant data from the original data, but also improve the classification accuracy of gene expression data sets.

## Conclusion and Discussion

Firstly, the concept of approximate conditional entropy is given and its monotonicity is proved in this article. Approximate conditional entropy can describe the uncertainty of knowledge from two aspects of boundary and information granule. And then, a novel feature selection algorithm FSACE is proposed based on the approximate conditional entropy. Finally, the effectiveness of the proposed algorithm is verified on several gene expression data sets. Experimental results show that compared with several state-of-the-art feature selection algorithms, the proposed feature selection algorithm not only can obtain compact features, but also improve classification performance. The time complexity of FSACE is *O*(|*U*|^2^|*C*|^2^). Because the gene expression data sets usually contain a large number of genes, the time complexity of FSACE is high. In addition, FSACE does not consider the interaction between attributes. Therefore, reducing the time complexity of FSACE and seeking more efficient feature selection algorithm considering interaction between attributes are two issues that we will study in the future.

## Data Availability Statement

Publicly available datasets were analyzed in this study. This data can be found here: http://portals.broadinstitute.org/cgi-bin/cancer/datasets.cgi (cancer Program Legacy Publication Resources).

## Author Contributions

The author confirms being the sole contributor of this work and has approved it for publication.

## Conflict of Interest

The authors declare that the research was conducted in the absence of any commercial or financial relationships that could be construed as a potential conflict of interest.
